# Microstructure Evolution and Mechanical Properties of Medium Carbon Martensitic Steel during Warm Rolling and Annealing Process

**DOI:** 10.3390/ma14226900

**Published:** 2021-11-15

**Authors:** Guolong Liu, Jingbao Liu, Jie Zhang, Minghe Zhang, Yunli Feng

**Affiliations:** 1 College of Metallurgy and Energy, North China University of Science and Technology, Tangshan 063210, China; lgl_1990@163.com (G.L.); hblg_yjxy@163.com (M.Z.); 2 Technology Center, HBIS Group Tangsteel Company, Tangshan 063016, China; tangsteel_1@sina.com (J.L.); tanggangsteel@sina.com (J.Z.)

**Keywords:** medium carbon martensitic steel, warm rolling, annealing, microstructure, mechanical properties

## Abstract

The microstructure evolution and mechanical properties of medium carbon martensitic steel during the warm rolling and annealing process were studied by scanning electron microscope (SEM), electron back scattering diffraction (EBSD), and electronic universal testing machine. The results showed that the microstructure of ferrite matrix with mass dispersive cementite particles was obtained by decomposition of martensitic in medium-carbon martensitic steel after warm rolling. The grain size of ferrite was ~0.53 μm, the yield strength and tensile strength were 951 MPa and 968 MPa, respectively, and the total elongation rate was 11.5% after warm rolling at 600 °C. Additionally, after the next 4 h of annealing, the grain size of ferrite and particle size of cementite increased to ~1.35 μm and ~360 nm and the yield strength and tensile strength decreased to 600 MPa and 645 MPa, respectively, with a total elongation increases of 20.9%. The strength of the material increased with increasing strain rate in tension, and the yield-to-tensile strength ratio increased from 0.92 to 0.94 and maintained good plasticity.

## 1. Introduction

Plain carbon steels have been widely used in engineering structural materials owing to their low cost, easy processing, and good mechanical properties [1]. In order to fulfill the increasingly stringent design requirements, the mechanical properties of carbon steels need to be continuously improved [2]. To improve the performance of carbon steels, researchers have devised many methods, including the addition of microalloying elements and microstructure control [3,4,5,6,7]. However, the production cost of microalloyed steel has increased due to the addition of alloying elements. Studies have confirmed that steels can simultaneously improve strength and plasticity by refining grains [8,9]. The severe plastic deformation (SPD) is the main method for grain refinement, which can refine the grain size of materials to ultra-fine or even nano-scale [10,11].

It was found that using quenched martensite as the initial microstructure of rolling deformation could obtain a better ultrafine grain structure [12]. At present, the research on obtaining ultra-fine-grained steel by cold plastic deformation of martensite mostly has focused on low carbon steels [13,14,15]. The characteristics of martensite structure are high hardness and large deformation resistance, which needs to combine with warm deformation to reduce the deformation resistance [16]. Poorganji et al. [12] concluded that the dynamic recovery and dynamic recrystallization of ferrite occurred in the process of warm deformation of the martensitic steel. During the heating and holding process, the fine carbides precipitated from the ferrite matrix played the role of pinning dislocations, which inhibits the grain growth and obtains significantly refined ferrite grains [17]. The yield strength of plain carbon steel is low and cannot be applied to high-strength structural components. It limits the application of carbon steel in high-strength moving parts, such as shaft parts, support transmission parts, fasteners, and other fields [18,19] Inspired by Lu et al., the microstructure of plain, medium carbon steel can be optimized without changing the composition, so as to obtain low-cost and high-performance alloy materials [20].

In this work, a plain medium carbon steel was selected to obtain a martensite structure by quenching. After warm rolling and annealing treatment, the multiphase structure, which consists of ultrafine ferrite and dispersed cementite, can be obtained. The process avoids the addition of alloy elements, improves the strength performance of the medium carbon steel, and makes it cost-effective and extended in industrial production. The microstructure of the plain medium carbon steel was characterized by scanning electron microscopy (SEM) and electron backscattered diffraction (EBSD). The effects of the warm rolling temperature and annealing time on the microstructure evolution, deformation behavior, and mechanical properties of medium carbon martensitic steel were expounded.

## 2. Materials and Methods

**Figure 1:**
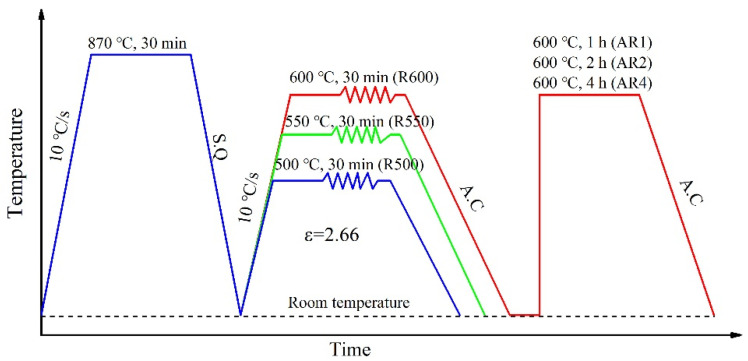
The pretreatment, warm rolling process, and heat treatment of the studied steel.

The experimental material was a plain medium carbon steel with 0.45% C, 0.54% Mn, 0.27% Si, 0.016% S, and 0.022% P (in mass). The samples were machined into a rectangular shape with the size of 20 mm × 20 mm × 200 mm for warm rolling. The specimens were pretreated in the KSW-5-12A box resistance furnace in a high-purity nitrogen atmosphere to prevent oxidation of the samples. Quenched samples were heated in a high-temperature, box-type furnace (Model: RX4-85-13), and then a six-passes rolling was carried out by a high-stiffness, four-roller, cold rolling mill (Model: Φ350 × 350 mm^2^). The total reduction amount of the sample was 90% and the strain was about 2.66. The pretreatment and warm rolling processes of the test steel are shown in Figure 1. The rolling temperature was 500 °C (R500), 550 °C (R550), and 600 °C (R600), respectively. After rolling, the samples were finally air-cooled to room temperature. The samples’ size of 2 mm × 5 mm × 100 mm (normal direction (ND) × transverse direction (TD) × rolling direction (RD)) was cut along the rolling direction for annealing by a wire cutting machine after warm rolling at different temperatures. The R600 samples were annealed in an annealing furnace at 600 °C for 1 h (AR1), 2 h (AR2), and 4 h (AR4), respectively.

The warm rolled plates and annealed plates were machined into samples of 2 mm × 5 mm × 10 mm for microstructure observation. Samples of warm rolling and annealing samples were etched with 4% nitric acid alcohol solution after grinding and mechanical polishing. Additionally, the RD × ND surfaces were examined by an FEI Quanta-650 FEG scanning electron microscopy (SEM, FEI, Hillsboro, OR, USA). The average particle size was measured by the Image-Pro Plus software (Version 6.0, Media Cybernetics, Silver Spring, MD, USA). Orientation and grain boundaries distribution of ferrite matrix after warm rolling and annealing were analyzed by electron backscattered diffraction (EBSD, AMETEK, Berwyn, PA, USA). The grain size analysis and the boundary analysis of each sample were all calculated from EBSD. After grinding and mechanical polishing, the electrolytic polishing was performed with the electrolyte, which was composed of anhydrous alcohol, high acid, and glycerol (16:3:1), and three 100 μm × 50 μm areas were scanned at different locations on each sample for statistical analysis. The tensile tests at room temperature were performed in WDW-ε50Ш electronic universal testing machines (Jinan Hensgrand Instrument Co., Ltd., Jinan, China) with 2.0 mm thickness, 6 mm width, and 32 mm length of reduced section (gauge length). The tensile axis was parallel to the rolling direction of the sample with a strain rate of 5 × 10^−4^ s^−1^. The different strain rate tensile tests were performed with AR2 specimens of 5 × 10^−1^ s^−1^, 5 × 10^−2^ s^−1,^ and 5 × 10^−3^ s^−1^, respectively. The tensile samples with different conditions were measured three times and their average values were calculated.

## 3. Results and Analysis

### 3.1. Martensitic Microstructure Evolution during Warm Rolling and Annealing

#### 3.1.1. SEM Observation and Analysis

**Figure 2:**
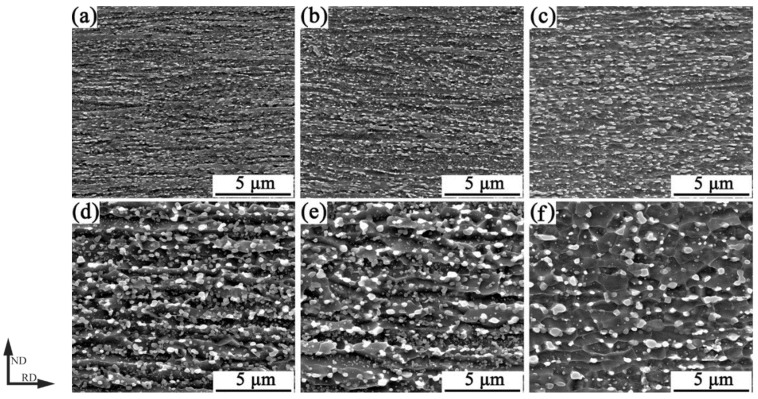
Micrographs of (**a**) 500 °C, (**b**) 550 °C, and (**c**) 600 °C warm rolling and warm rolled at 600 °C annealing at 600 °C for (**d**) 1 h, (**e**) 2 h, and (**f**) 4 h.

Figure 2 shows the SEM images of the medium carbon martensitic steel after warm rolling and annealing. From Figure 2a–c, it can be seen that the microstructures after warm rolling were ferrite matrices and dispersed cementite particles. When the rolling temperature was 500 °C, the cementite particles were mainly spherical and small-size short bars, as shown in Figure 2a. When the rolling temperature rose to 550 °C, the size of the cementite particles increased, and the fraction of the spherical cementite particles increased, as shown in Figure 2b. When the rolling temperature reached 600 °C, the size of the cementite particles further increased, as shown in Figure 2c. As the rolling temperature increased, the average grain size of ferrite increased. Calculated by Image-Pro Plus at rolling temperatures of 500 °C, 550 °C, and 600 °C, the average grain sizes were found to be 0.41 μm, 0.44 μm, and 0.53 μm, respectively.

From Figure 2d–f, it can be seen that, after annealing and holding for 1 h, only part of the ferrite recrystallized to form fine, equiaxed grains after, but most of the ferrite grains still exhibited an elongated state along the rolling direction. The average ferrite grain size was significantly larger than that after warm rolling, as shown in Figure 2d. As the annealing holding time was extended to 2 h, the ratio of ferrite reversion and recrystallization increased, and the fraction of equiaxed ferrite grains increased. However, there were still some ferrite grains in an elongated state, and the average ferrite grain size increased, as shown in Figure 2e. After extending the annealing holding time to 4 h, the majority of ferrite grains recrystallized with an increase of the fraction of equiaxed ferrite grains, a decrease of the fraction of cementite particles in ferrite grains, and a significant enlarged carburized grains at grain boundaries, as shown in Figure 2f. Compared with the warm rolled samples, the average grain size of ferrite increased due to recrystallization and grain growth during annealing. When annealed at 600 °C for 1 h, 2 h, and 4 h, the grain sizes of the studied steel were 0.82 μm, 1.17 μm, and 1.35 μm, respectively. It is well known that the pinning effect is related to the size and volume fraction of particles [21]. As the extension of the annealing holding time, the size of cementite particles increased, and the pinning effect decreased, which led to an increase in grain boundary migration and ferrite grains’ growth.

**Figure 3:**
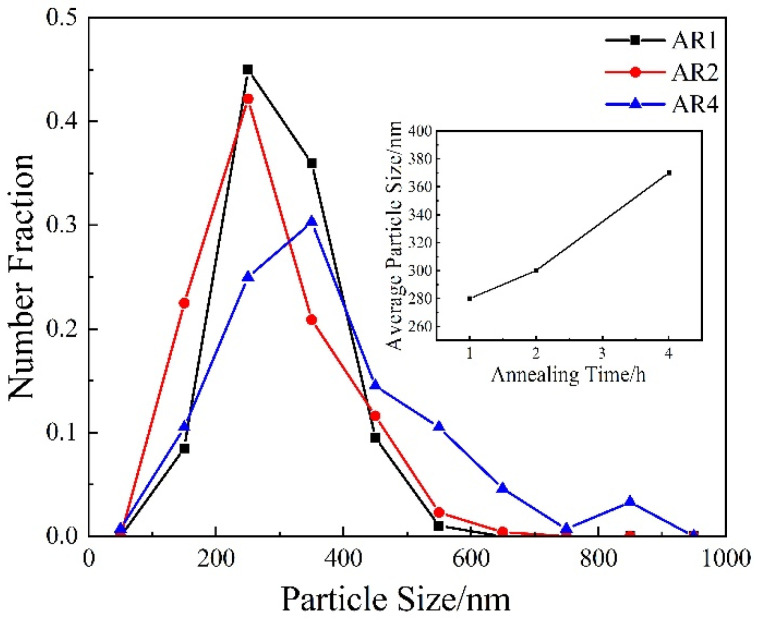
The distribution of cementite particle sizes after annealing at 600 °C for different times.

Figure 3 shows the distribution of cementite particle sizes after annealing at 600 °C for a different time. It can be seen that the particle size of cementite changed with the extension of the annealing time. When the annealing time was 1 h, the cementite particle size was mostly concentrated between 150 and 400 nm, and the average grain size was close to 280 nm. When the annealing time was 2 h, the size was mostly concentrated between 200 and 400 nm, and the average grain size approached 300 nm. When the annealing time was 4 h, the particle size of cementite was dispersed, and the fraction of large cementite particles increased significantly, with an average size of 370 nm. At all stages of tensile strain, fine cementite particles are distributed in grain boundaries or within the grains, which will effectively hinder dislocation slippage. In addition, the fine intragranular cementite particles contribute to the density of geometrically necessary dislocations(ρ^G^) so it can weaken the dynamic recovery effect, which decreases the ρ^G^. Therefore, during the tensile deformation, in the high-strain stage, the additional hardening caused by the fine dispersed cementite particles reduced the rate of decrease of the work hardening rate, thereby allowing the annealed samples to obtain a greater uniform elongation.

#### 3.1.2. EBSD Observation and Analysis

**Figure 4:**
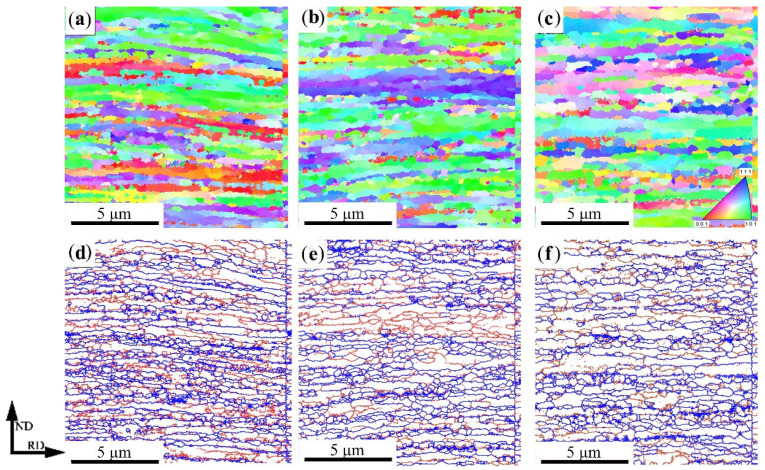
EBSD orientation map of (**a**) 500 °C, (**b**) 550 °C, and (**c**) 600 °C warm rolling samples and EBSD boundary map of (**d**) 500 °C, (**e**) 550 °C, and (**f**) 600 °C warm rolling samples.

Figure 4 shows the EBSD maps of medium carbon martensite steel after warm rolling at different temperatures, where Figure 4a–c is the orientation distribution of grains and Figure 4d–f is the boundaries’ distribution in which the blue and red lines represent the high-angle grain boundaries (HAGBs) (θ≥15°) and low-angle grain boundaries (LAGBs) (2≤θ≤15°), respectively. In Figure 4, it can be seen that the microstructure had obvious orientation and the ferrite grains were distributed in a band along the rolling direction. When rolled at 500 °C, <001> and <101> were the main grain orientations. As a result of the dynamic recrystallization of part of the banded ferrite, the fine equiaxed ferrite grains appeared in the local areas and were surrounded by LAGB. As the rolling temperature increased, the grains near the <001> orientation gradually decreased. The grain orientation was mainly <111> and <101> when the rolling temperature was 550 °C, but when it rolled at 600 °C, part of the ferrite grains near the orientations of the <111> and <101> transformed into a new ferrite grain with the orientation near <112>. As the rolling temperature increased, the amount of ferrite with dynamic recovery and recrystallization increased. At the same time, the amount of banded ferrite grains was reduced. During the rolling process, the fine, equiaxed ferrite grains merged with each other and grew as the grain boundaries moved. The size of equiaxed ferrite grains gradually increased, and the grain size tended to be more uniformly distributed.

**Figure 5:**
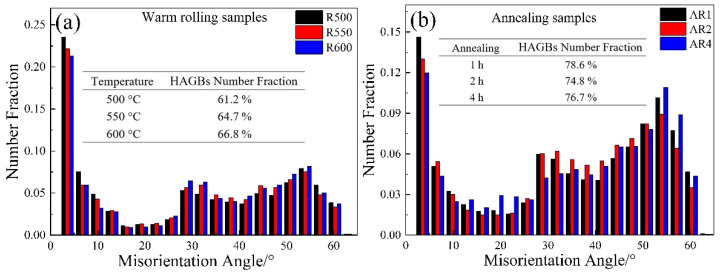
The grain boundary misorientation distribution: (**a**) warm rolling samples and (**b**) annealing samples.

The misorientation distribution of ferrite grains at different rolling temperatures is shown in Figure 5a. It can be seen that when the rolling temperature was 500 °C, more sub-grain boundaries appeared in the banded ferrite grains. The proportion of HAGBs was about 61.2%, and, as the rolling temperature increased, the sub-grain boundary gradually became a HAGB. When the rolling temperature was 550 °C and 600 °C, the proportion of HAGB was 64.7% and 66.8%, respectively, and the boundary angle was mainly distributed between 30° and 55°. During the warm rolling process, due to the increase of the rolling temperature, the fraction of dynamic recrystallization of ferrite and equiaxial ferrite grains increased, so the fraction of HAGBs increased with the increase of the rolling temperature. After annealing the R600 sample, the fraction of HAGBs was about 75.0%. When the samples were annealed for 1 h, the recrystallization was completed.

### 3.2. Mechanical Properties of Martensitic Steel after Warm Rolling and Annealing

**Figure 6:**
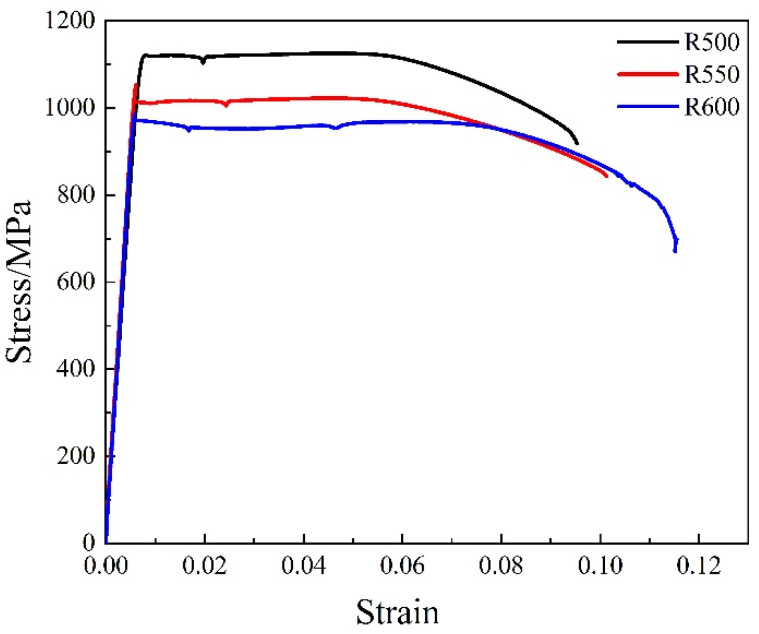
Stress–strain curve of samples warm rolled at different temperature.

The stress-strain curves of warm rolling at different temperatures are shown in Figure 6. When the rolling temperature was 500 °C, the yield strength and tensile strength of the samples were 1108 MPa and 1126 MPa, respectively. When the rolling temperature was 550 °C and 600 °C, the yield strength of the samples was 1005 MPa and 951 MPa, respectively, and the tensile strength was 1022 MPa and 968 MPa, respectively. The results showed that the yield strength and tensile strength decreased as the rolling temperature increased, but the tensile strength decreased more slowly. The ratio of the yield strength to the tensile strength of the sample after rolling was about 0.98. When the yield-to-tensile strength ratio was close to 1, the samples’ necking happened immediately after yielding during the tensile test at room temperature, and the work hardening ability was low. In addition, as the rolling temperature increased, the elongation increased; the total elongation was 9.5%, 10.1%, and 11.5%, respectively, when the rolling temperature was 500 °C, 550 °C, and 600 °C, and the average elongation was about 3% to 6%.

When the samples were rolled at 500 °C, the lath martensite had higher internal stress and showed more LAGBs, resulting in higher hardness and strength and lower total elongation. As the rolling temperature increased, the internal stress in the hard brittle martensite phase remaining in the microstructure decreased, and a dynamic reversion occurred. On the other hand, the size of the cementite particles increased, as shown in Figure 2a–c. Therefore, the pinning effect on dislocations and grain boundaries was reduced so that the hardness and strength were reduced while the total elongation increased.

**Figure 7:**
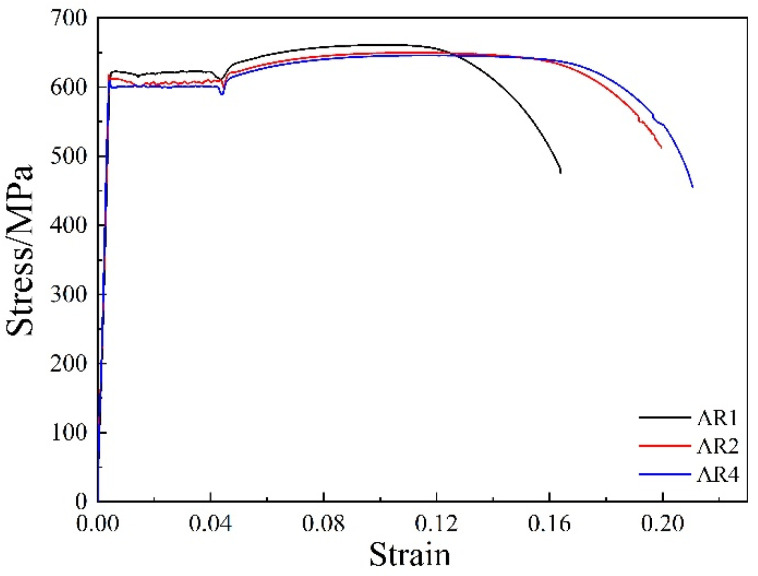
Stress–strain curve of samples annealed at 600 °C for different time.

The stress-strain curves of the R600 samples after annealing at 600 °C are shown in Figure 7. The yield platform is clearly present in the tensile curve. Lee. T et al. [22] found that the appearance of the yield point is related to the reduction of the work hardening rate and dislocation slip. As the grain size decreases, the grain boundaries increase, which provides more sources of dislocations. Since dislocations accumulate at the ferrite grain boundaries, dislocations in adjacent ferrite grains are activated and start to move. In the initial stages of plastic deformation, dislocations should break away from the pinning of carbon and other interstitial atoms to form a clear yield point. When the annealing time was increased from 1 h to 4 h, the yield strength and tensile strength decreased from 614 MPa and 660 MPa to 600 MPa and 645 MPa. The uniform elongations were 10.5%, 12.2%, and 12.5%, respectively, and the total elongations were 16.4%, 19.9%, and 20.9%, respectively. The yield-to-tensile strength ratio of all annealed samples was approximately 0.93, and the total elongation was greater than 16%. The higher yield-to-tensile strength ratio was due to the uniform distribution of carbides [23,24]. As the heat preservation time increased, the fraction of recrystallized ferrite grains gradually increased and the grain size increased, while the density of defects such as dislocation in the microstructure decreased. The hardening effect of the process was weakened, and the plasticity of the material was increased. With the holding time increased, the yield strength and tensile strength gradually decreased, and the uniform elongation and total elongation increased.

The annealed microstructure of the medium carbon martensitic steel after warm rolling was a multi-sized grain structure alternating between coarse- and fine-grain layers. Under the action of tensile load, the coarse grain layer began to plastically deform due to its small plastic deformation resistance, and then gradually transformed into a fine-grain layer with greater resistance. In this way, strain fit will occur between layers of different grain sizes within the material. Since the interface between the different layers resulted in a strain continuity, the non-uniform deformation between the different layers resulted in the strain gradient between the layers. In addition, the strain incompatibility between layers of different grain sizes may transform the uniaxial stress state into a multiaxial stress state during uniaxial tension, which will activate more slip systems to initiate and strengthen the interaction and entanglement of dislocations [25]. Therefore, the strain gradient caused by the multimodal distribution of grain size and the multiaxial stress state promoted the accumulation and storage of dislocations. The annealed sample exhibited a stable uniform deformation stage, and its uniform elongation and the total elongation during the tensile process were significantly improved. Furthermore, as described above, the fine dispersion of cementite particles on the ferrite matrix can increase the uniform elongation.

**Figure 8:**
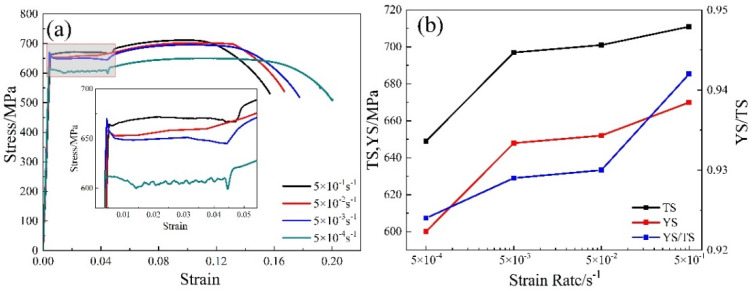
Stress–strain curve (**a**) and intensity change (**b**) of annealed samples under different strain rate of AR2.

The AR2 samples were subjected to tensile tests at different strain rates, which were 5 × 10^−1^ s^−1^, 5 × 10^−2^ s^−1^, 5 × 10^−3^ s^−1^, and 5 × 10^−4^ s^−1^, respectively, at room temperature. The stress-strain curves are shown in Figure 8a. In the stress-strain curve, there was a significant sawtooth fluctuation at a strain rate of 5 × 10^−4^ s^−1^ on the yield platform. The stress tended to stabilize, the yield strength was significantly reduced, and the strain rate was increased to 5 × 10^−3^ s^−1^. When the strain rate increased to 5 × 10^−2^ s^−1^, with the increase of the stress, the significant lower concave tendency yield disappeared. When the strain rate rose to 5 × 10^−1^ s^−1^, the yield decreased significantly. The strength of the material was determined by an internal variable, that is, dislocation density, and an external variable, that is, strain rate [26]. After annealing at different tensile rates, which were between 5 × 10^−4^ s^−1^ and 5 × 10^−1^ s^−1^, the samples had similar dislocation density, which determined the hardening pattern. Plastic strain was localized to high dislocation density/low strain rate (forest hardening zone) or low dislocation density/high strain rate (strain rate hardening zone) [26,27,28,29]. During the tension process, local hardening led to non-uniform plastic strain patterns. With the gradual increase of tensile stress, a small number of original dislocations continued to strain, and a large number of dislocations accumulated on the slip surface. These dislocations further affected the deformation process, forming significant slip bands, resulting in significant sawtooth fluctuation stress [26]. These dislocations, which led to significant sawtooth fluctuations of stress, formed obvious slip bands during the affected deformation process. In the case of moderate dislocation density/moderate strain rate deformation, the strain rate strengthening mode will transform to forest hardening mode. During the deformation of moderate dislocation density/moderate strain rate, the strengthening mode changed from strain rate hardening to forest hardening. Under the synergistic effect of forest hardening and strain rate strengthening, the plastic strain in the transition zone between the two regions was the most uniform [29]. The uniform deformation mode was caused by the continuous fracture and realignment of the dislocation joints.

It can be seen from Figure 8b that the yield strength and tensile strength increased rapidly as the strain rate increased from 5 × 10^−4^ s^−1^ to 5 × 10^−1^ s^−1^, then the increase turned slow. The yield-to-tensile strength ratio increased with the increase of the strain rate, which had a significant effect on the yield strength. When the strain rate increased from 5 × 10^−4^ s^−1^ to 5 × 10^−1^ s^−1^, the yield strength increased from 600 MPa to 670 MPa, and the tensile strength increased from 649 MPa to 711 MPa, increasing by 70 MPa and 62 MPa, respectively. The yield-to-tensile strength ratio increased from 0.92 to 0.94. Correspondingly, the elongation decreased as the strain rate increased. When the strain rate was 5 × 10^−4^ s^−1^ and 5 × 10^−1^ s^−1^, the uniform elongation and total elongation were 12.4%, 10.3%, 20.0%, and 15.7%, respectively. Since dislocation movement and dislocation increment are the essence of plastic deformation, the slippage of dislocation requires a certain amount of time. The greater the tensile strain rates at room temperature, the greater the speed of dislocation increment and motion during plastic deformation, and the greater the time-delay effect. The crystal rotation in the direction of the external force is not sufficient, and the formation and extension of the slip bands are blocked. Therefore, the deformation resistance and tensile strength are increased, whereas the elongation decreases as the strain rate increases [30,31].

When used as a connector, the annealed sample had a higher yield strength and good performance. The mechanical properties of the annealed warm-rolled samples reached the mechanical property standards of certain high-strength, low-alloy steels [32], which is expected to replace the use of certain high-strength, low-alloy steels. Therefore, reducing the use of alloying elements can reduce industrial production costs and expand the application of plain, medium carbon steels [5].

## 4. Conclusions

(1) After warm rolling at 500 °C, 550 °C, and 600 °C, the martensite of medium carbon martensite steel was decomposed. When warm-rolled at 500 °C, 550 °C, and 600 °C, the average ferrite grain size was 0.41 μm, 0.44 μm, and 0.53 μm, respectively, and the proportion of HAGBs was 61.2%, 64.7%, and 66.8%, respectively. The room temperature tensile strength of the warm-rolled samples was above 950 MPa, accompanied by the total elongation of about 10%, which is of great value for application to a certain extent.
(2) The samples were annealed at 600 °C after warm rolling. As the holding time increased, the fraction of cementite particles in the grains decreased, and the size of cementite particles at the grain boundaries increased. The yield strength of the annealed samples was above 600 MPa, and the total elongation was above 16%, which had a good combination of strength and plasticity.
(3) The yield-to-tensile strength ratio of the samples after warm rolling and annealing at 600 °C for 2 h at different strain rates was greater than 0.92, and it had a high yield strength, that is, greater than 600 MPa, which had good performance when used as a fastener.

## Data Availability

No new data were created or analyzed in this study. Data sharing is not applicable to this article.
